# Hospital Admission to a Window-Side Bed Does Not Prevent Delirium: A Retrospective Cohort Study of Older Medical Inpatients in General Wards

**DOI:** 10.3389/fmed.2021.744581

**Published:** 2021-09-14

**Authors:** Daiki Aomura, Yosuke Yamada, Makoto Harada, Koji Hashimoto, Yuji Kamijo

**Affiliations:** Department of Nephrology, Shinshu University School of Medicine, Matsumoto, Japan

**Keywords:** delirium, general ward, internal medicine, older patients, window-side bed

## Abstract

**Background:** Delirium in older inpatients is a serious problem. The presence of a window in the intensive care unit has been reported to improve delirium. However, no study has investigated whether window-side bed placement is also effective for delirium prevention in a general ward.

**Objectives:** This study aims to clarify the association between admission to a window-side bed and delirium development in older patients in a general ward.

**Design:** This research is designed as a retrospective cohort study of older patients admitted to the internal medicine departments of Shinshu University Hospital, Japan.

**Participants:** The inclusion criteria were the following: (1) admitted to hospital internal medicine departments between April 2009 and December 2018, (2) older than 75 years, (3) admitted to a multi-patient room in a general ward, and (4) unplanned admission. The number of eligible patients was 1,556.

**Exposure:** This study is a comparison of 495 patients assigned to a window-side bed (window group) with 1,061 patients assigned to a non-window-side bed (non-window group). When patients were transferred to the other type bed after admission, observation was censored.

**Main Measures:** The main outcome of interest was “delirium with event” (e.g., the use of medication or physical restraint for delirium) within 14 days after admission as surveyed by medical chart review in a blinded manner.

**Key Results:** The patients had a median age of 80 years and 38.1% were female. The main outcome was recorded in 36 patients in the window group (10.7 per thousand person-days) and 84 in the non-window group (11.7 per thousand person-days). Log-rank testing showed no significant difference between the groups (*p* = 0.78). Multivariate analysis with Cox regression modeling also revealed no significant association for the window group with main outcome development (adjusted hazard ratio 0.90, 95% confidence interval of 0.61–1.34).

**Conclusions:** Admission to a window-side bed did not prevent delirium development in older patients admitted to a general ward.

## Introduction

Characterized by an acute disturbance in mental abilities resulting in confusion and abnormal behavior (1), delirium is a serious problem in older inpatients. Although delirium develops the most in inpatients admitted to the intensive care unit (ICU) or after surgery, older inpatients with medical diseases were also reported to exhibit delirium at rates of 6–26% (2). The condition harms patients by increasing mortality risk during hospitalization, prolonging hospital stay, and diminishing independence and cognitive function after discharge (3–8). Furthermore, delirium imposes considerable financial costs on health care systems (9).

To prevent or treat delirium, non-pharmacologic multicomponent approaches are highly encouraged, including orientation to time and place, cognitive stimulation, early mobilization, sleep enhancement, identification of underlying delirium causes, detecting early signs of delirium, and educating nursing staff and family members (10–12). However, those approaches can be labor intensive (12) and are difficult to perform for large numbers of inpatients admitted into general wards. Pharmacological approaches with neuroleptics and sedative medicines are also employed to control the symptoms of delirium. However, no convincing evidence supports the use or effect of any drug against delirium (13), with some studies even reporting potential harm (14, 15). Further prophylactic and therapeutic strategies are needed for general wards.

Delirium in patients on a general ward may be prevented and improved by assignment to a window-side bed, with circadian regulation by exposure to sunlight, phototherapy, and melatonin agonists being reportedly effective (16–18). As patients in window-side beds receive considerably more direct exposure to sunlight, their circadian rhythm may be better regulated to help prevent delirium. Furthermore, visibility to the outside through a window could suppress delirium by maintenance of cognition (19, 20). Indeed, several studies have described that the presence of a window in the inpatient room is effective for managing delirium in the ICU (21, 22), and several expert opinions recommend placing delirious patients near a window (23, 24). Considering that window-side bed assignment bears no additional costs or labor requirements and causes no side effects, this management strategy may be a simple and effective approach against delirium.

To date, no study has focused on the effect of windows against delirium in general wards, and the impact of window-side placement has not been addressed. This study examined the association of admission to a window-side bed with delirium development in older inpatients in a general hospital ward.

## Methods

### Study Design

This was a retrospective cohort study reviewing the medical charts of patients admitted to Shinshu University Hospital, Japan.

### Setting and Study Population

The inclusion criteria of this investigation were as follows: (1) admitted to any internal medicine department at Shinshu University Hospital, Japan, between April 2009 and December 2018, (2) older than 75 years, (3) admitted to a multi-patient room in a general ward, and (4) unplanned admission. Only patients in the internal medicine departments were included to eliminate the effect of operations on delirium. Patients with scheduled admission, including those for a medical check-up, were excluded to identify patients at a higher risk of delirium. The exclusion criteria were as follows: (1) transfer from another hospital, (2) regularly taking a medicine for delirium before admission, and (3) already delirious on admission. Patients satisfying exclusion criterion (1) were excluded due to a lack of data, while those meeting criteria (2) or (3) were dropped since they might have already been delirious at admission. Medication for delirium was defined as any antipsychotics, mianserin, trazodone, or yokukansan which have all been generally prescribed for delirium (13, 25–27). Delirious at admission was defined as already exhibiting “delirium with event” which was the main outcome of this study (described below), within 3 h after admission.

### Baseline Characteristics

The data of eligible patients were collected from hospital medical records and included bed position at admission, transfer to another bed during hospitalization, basic clinical information, sequential organ failure assessment (SOFA) score (28, 29), performance status (PS) (30), Charlson comorbidity index (CCI) (31), daily medicine use before admission, pre-existing dementia, and main disease for admission. To calculate SOFA score, blood test data obtained within 24 h after admission was referred and the partial pressure of oxygen in arterial blood from peripheral oxygen saturation was predicted using the Hill equation (32). The PS scores were obtained as assessed by nurses on patient admission. The CCI, pre-existing dementia, and the main disease for admission were ascertained using the records of the registered disease name on admission. The main disease for admission was classified as a central nervous system disorder, cardiovascular disease, infection, malignancy, or others.

### Exposure of Interest

**Figure 1:**
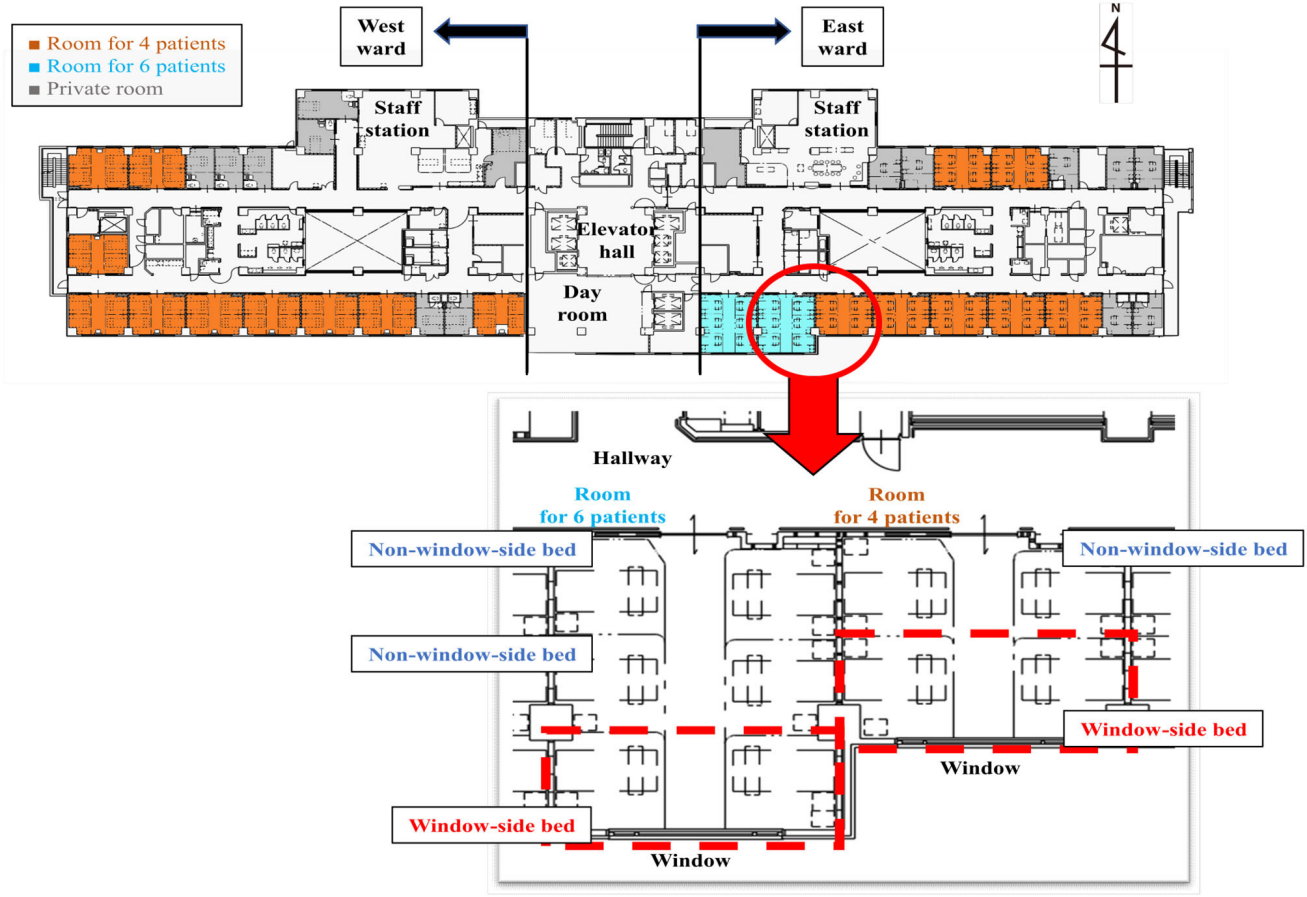
Map of a general hospital ward. The map shows the layout of a general ward at Shinshu University Hospital. There are two types of multi-patient rooms: rooms for four patients and rooms for six patients. In both room types, the two beds nearest to the window are designated window-side beds (red dotted lines), while the others are designated non-window-side beds. Each bed is separated by a dividing curtain.

Eligible patients were divided into the group admitted to a window-side bed (window group) and the group admitted to a non-window-side bed (non-window group). At Shinshu University Hospital, multi-patient rooms in general wards can accommodate up to four or six patients. In rooms for four patients, the two beds closest to the window were defined as window-side beds and the two remaining beds were considered non-window-side beds (Figure 1). In rooms for six patients, the two beds closest to the window were judged as window-side beds and the remaining four beds were defined as non-window-side beds. Each bed was separated by a dividing curtain. Bedside luminosity was measured to confirm the hypothesis that patients in window-side beds received more exposure to natural light. The luminosity of each of the two beds at window-side beds and non-window-side beds in rooms facing south and north were determined with a luminometer (EM-9300SD, SATOSHOJI, Japan) every 3 h from 9:00 A.M. to 9:00 P.M. on March 30, 2019, on a clear day on the seventh floor of the hospital.

### Outcome Assessment

The primary outcome was “delirium with event” within 14 days after admission. The definition and abstraction method of this outcome are as follows. First, two physicians reviewed the medical charts of eligible hospitalized patients and identified delirium development using a chart-based method for identification (33). In this method, the physicians searched for key terms indicating acute mental change (e.g., delirium, mental state change, inattention, disorientation, hallucinations, agitation, inappropriate behavior, etc.). If the acute mental change could not be explained by reasons other than delirium (e.g., central nervous system disorder or dementia), the patient was defined as having “delirium.” To enhance the reproducibility and specificity of the outcome, physicians further assessed whether the abstracted “delirium” was accompanied by any of the following events: (1) use of any drugs as sedatives for delirium, including antipsychotics, mianserin, trazodone, yokukansan, benzodiazepines, and first-generation antihistamines, (2) physical restraint, (3) transfer to another bed, (4) transient stay in the staff room for monitoring, and (5) self-removal of drip line or catheter (34, 35). If the delirium was accompanied by any such event, the case was classified as “delirium with event.” The observation period for the primary outcome was limited to 14 days after admission to exclude the influence of a long hospital stay on delirium. Fourteen days was also chosen since Japanese medical staff are basically recommended to discharge patients within 14 days considering that some medical fees are covered by national healthcare for only 14 days of admission. Both physicians reviewed the medical charts independently and were blinded to whether the patient was in the window or non-window group. If their judgment differed on an outcome, mutual consensus was reached by discussion.

The secondary outcomes of “delirium with event” was also assessed within 30 days after admission, “delirium” within 14 or 30 days after admission, hospital stay longer than 14 days, transfer to the ICU, and death during hospitalization.

### Sample Size Decision

Previous literature suggested the primary outcome to occur at a frequency of approximately 10% (2). To detect an absolute difference of 5% in the ratio of the primary outcome between the two groups (i.e., 7.5% in the window group and 12.5% in the non-window group) with 80% power at a 5% significance level, a total of 1,280 patients (divided at a 2:3 ratio) were required. Based on this calculation, the final recruitment target was set at 1,500 patients.

### Statistical Analysis

Descriptive statistics were employed to summarize the demographic factors of the patients stratified by two groups. Continuous variables were presented as the median and interquartile range (IQR) and compared using the Wilcoxon–Mann–Whitney test. Categorical variables were presented as the number and percentage and assessed by means of the chi-square test.

At the assessment of “delirium with event” or “delirium,” observation was censored when the following events were recorded for reasons other than delirium: (1) use of antipsychotics, mianserin, trazodone, or yokukansan, (2) physical restraint, (3) transfer to another bed (apart from window-side bed to window-side bed or non-window-side bed to non-window-side bed), (4) transient stay in the staff room for monitoring, and (5) self-removal of drip line or catheter. Kaplan–Meier curves of cumulative outcome incidence were calculated and compared between the groups using the log-rank test. The hazard ratio (HR) of the window group for the main outcome was estimated using multivariable Cox proportional hazard models to adjust for such potential confounders as age, sex, low body weight (i.e., body mass index less than 18.5), SOFA score, regular use of risk drugs for delirium before admission (e.g., benzodiazepines, non-benzodiazepines, anti-histamines, and narcotic analgesics), PS, CCI, admission for central nervous system disorders, and pre-existing dementia (36–38). Concerning the assessments of hospital stay for longer than 14 days, transfer to the ICU, and death during hospitalization, the observation period was limited not to 14 days, but to the entire time of hospitalization, and censoring was not taken into account. The adjusted odds ratios of the window group for those outcomes were estimated using logistic regression models. Additional subgroup analyses were conducted using various factors related to: (1) the environment of the inpatient and bed, including the type of room, direction of ward, direction of room and window, and season of admission, and (2) patient characteristics including age, sex, low body weight, SOFA score, regular use of risk drugs for delirium before admission, PS, CCI, admission for central nervous system disorders, and pre-existing dementia. The adjusted HR of the window group was assessed for the primary outcome in each subgroup. Each subgroup factor was excluded from its own regression model (e.g., age was excluded from the regression model in the subgroup analysis relating to age). Regarding age, SOFA score, PS, and CCI, the patients were divided into subgroups according to median values.

Multiple imputation was performed to account for missing data values for PS and SOFA scores in 269 patients. Each missing value was replaced with a set of substituted plausible values by creating 20 filled-in complete data sets by multiple imputation using a chained equation method (39). To test the robustness of the results with the multiple imputation method, complete case analysis and median imputation analysis were also performed as sensitivity analyses regarding the assessment of the main outcome.

All statistical analyses were performed using IBM SPSS statistics version 27.0 (IBM, Armonk, NY). Values of *p* < 0.05 were considered statistically significant.

### Ethics Approval and Consent to Participate

This study followed the reporting guidelines of Strengthening the Reporting of Observational Studies in Epidemiology. It was performed in accordance with the tenets set forth in the Declaration of Helsinki and approved by the ethics committee of Shinshu University Hospital (authorization number: 4329). Informed written consent was waived in this study by the ethics committee of Shinshu University Hospital due to its retrospective nature using medical records that did not subject the patients to new interventions. The collected data were anonymously stored and used for analysis. As an alternative to written informed consent, an opt-out document was created and posted on the hospital website that contained information on the design of the research and publication of the results to provide subjects the opportunity to halt the provision of their medical data.

## Results

### Bedside Luminosity

From 9:00 A.M. to 3:00 P.M, bedside luminosity was considerably higher at window-side beds (~600–1,100 lux) than at non-window-side beds (~300–400 lux), regardless of whether the room faced south or north (data not shown). Luminosity was undetectable at 9:00 P.M., after lights-out. These results strongly implied that the patients of window group received much more natural light cycle than those of the non-window group.

### Baseline Characteristics

**Figure 2:**
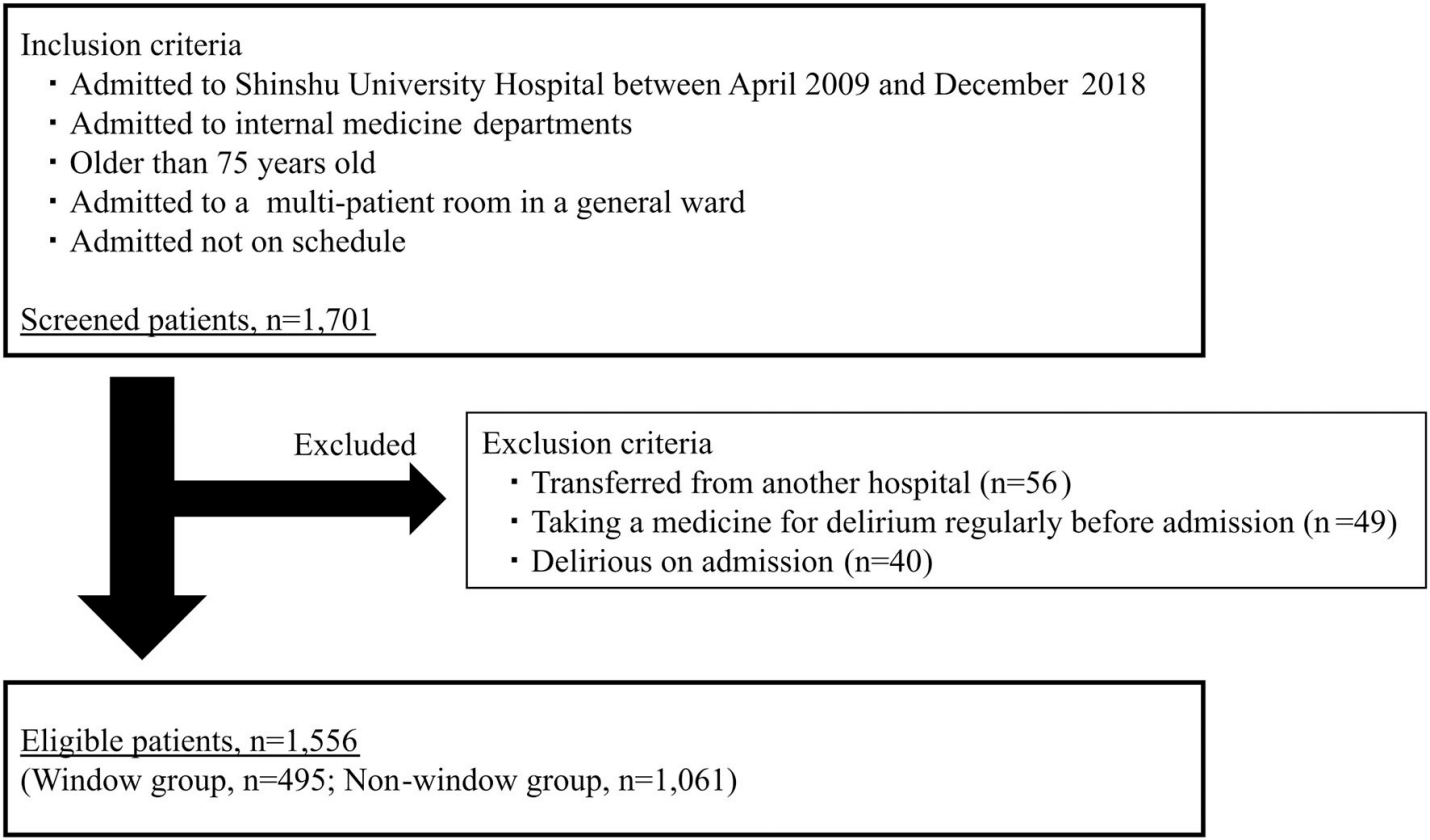
Study flow chart. Inclusion and exclusion criteria of this study. The number of eligible patients was 1,556.

The number of patients fulfilling the inclusion criteria was 1,701, among which eligible subjects totaled 1,556 after the exclusion of 145 patients (Figure 2). Regarding the characteristics of the eligible patients, median age was 80 years (IQR 77 to 84) and the proportion of female was 38.1%. All patients were Japanese. The characteristics of the patients in the window group (*n* = 495) and non-window group (*n* = 1,061) are presented in Table 1. There were no significant differences between the groups for basic characteristics or physical condition, such as SOFA score, PS, or CCI. The characteristics of patients with and without missing data differed significantly for age (median age: 79 and 80 years, respectively, *p* = 0.03) and admission with cardiovascular disease (16.7 and 11.6%, respectively, *p* = 0.02).

**Table 1:** Baseline cohort characteristics.

	**Window group**	**Non-window group**	***p*-value**
	**(*n* = 495)**	**(*n* = 1,061)**	
Age, median [IQR^*^], y	80.0 [77.0, 84.0]	80.0 [77.0, 84.0]	0.32
Female, *n* (%)	183 (37.0)	410 (38.6)	0.54
Body mass index, median [IQR^*^]	21.0 [18.8, 23.6]	21.3 [19.0, 23.7]	0.34
SOFA^†^ score, median [IQR^*^]	2.0 [1.0, 3.0]	2.0 [1.0, 3.0]	0.98
Missing, *n* (%)	16 (3.2)	41 (3.9)	0.66
Regular drug use before admission, *n* (%)			
Benzodiazepines	99 (20.2)	208 (19.9)	0.89
Non-benzodiazepines	36 (7.4)	97 (9.3)	0.24
Anti-histamines	46 (9.4)	88 (8.5)	0.56
Narcotic analgesics	21 (4.3)	20 (1.9)	0.01
Any of the above drugs	179 (36.2)	349 (32.9)	0.21
Performance Status, median [IQR^*^]	0.0 [0.0, 2.0]	0.0 [0.0, 1.0]	0.57
Missing, *n* (%)	59 (11.9)	162 (15.3)	0.09
CCI^‡^, median [IQR^*^]	2.0 [1.0, 3.0]	2.0 [1.0, 3.0]	0.78
Type of main disease, *n* (%)			
Central nervous system disorder	38 (7.7)	57 (5.4)	0.09
Cardiovascular disease	64 (12.9)	130 (12.3)	0.74
Infection	79 (16.0)	125 (11.8)	0.02
Malignancy	73 (14.7)	166 (15.6)	0.71
Other	241 (48.7)	583 (54.9)	0.02
Pre-existing dementia	13 (2.6)	27 (2.5)	1.00

* *IQR, interquartile range;*

† *SOFA, sequential organ failure assessment;*

‡ *CCI, Charlson comorbidity index*.

### Association of Window Group With Primary Outcome

**Figure 3:**
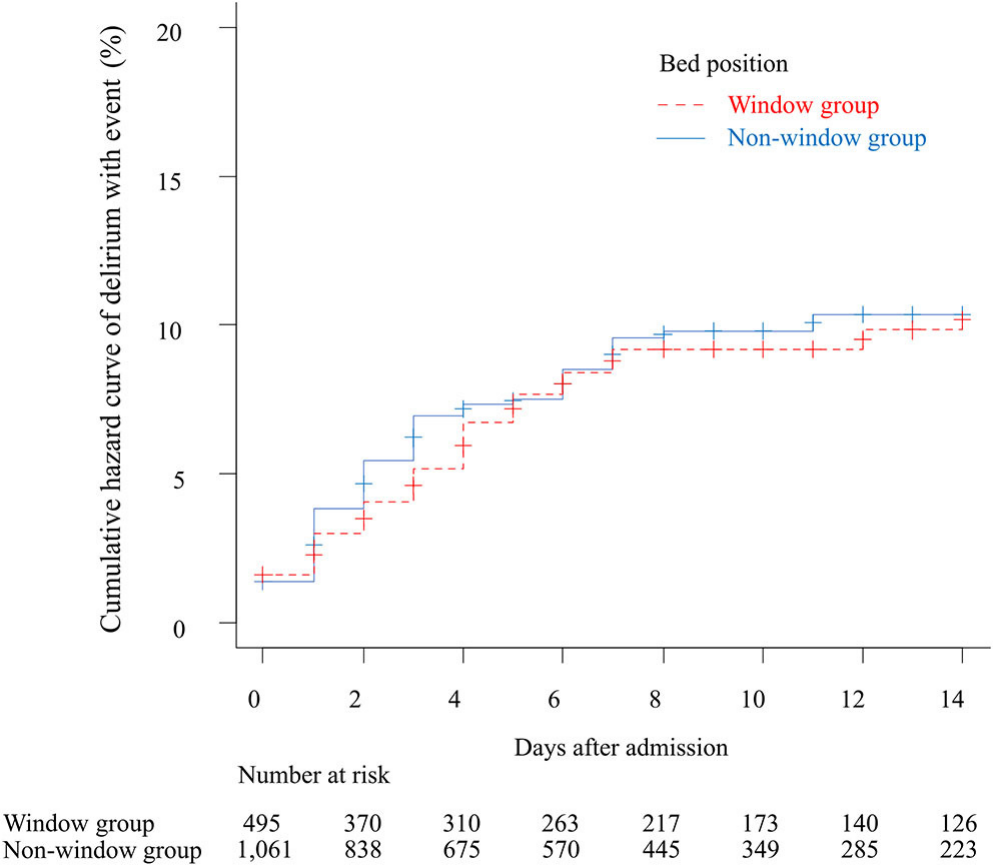
Unadjusted cumulative curves for the primary outcome. The figure shows the cumulative curves of delirium with event within 14 days after admission. The dotted red line indicates the window group and the solid blue line indicates the non-window group.

The incidence of “delirium with event” within 14 days after admission was 120 patients (7.7%; 11.4 per thousand person-days), and the breakdown of events was as follows: use of drugs for delirium in 56 cases (46.7%), physical restraint in 37 cases (30.8%), transfer to another bed in 12 cases (10%), transient stay in the staff room in 10 cases (9.8%), and self-removal of drip line or catheter in 4 cases (3.3%). The primary outcome was recorded in 36 cases in the window group (10.7 per thousand person-days) and in 84 cases in the non-window group (11.7 per thousand person-days). The unadjusted cumulative hazard curves for the primary outcome in the window and non-window groups are shown in Figure 3. Log-rank testing did not identify any remarkable difference between the groups (*p* = 0.78). Multivariate analysis with Cox regression models revealed no significant associations for the window group with the primary outcome [adjusted HR 0.90, 95% confidence interval (CI) 0.61–1.34, *p* = 0.62] (Table 2). The results of sensitivity analyses on missing data cases were similar for complete case analysis (adjusted HR 1.06, 95% CI 0.71–1.60, *p* = 0.77) and median imputation analysis (adjusted HR 0.89, 95% CI 0.60–1.32, *p* = 0.56).

**Table 2:** Multivariable analysis on the association of window group and delirium with event.

	Hazard ratio (95% CI^*^)	***p*-value**
Crude model	0.95 (0.64–1.40)	0.78
Adjusted model 1	0.96 (0.65–1.42)	0.84
Adjusted model 2	0.90 (0.61–1.34)	0.62

*Table shows the hazard ratio of the window group for delirium with event within 14 days after admission by Cox regression models. In model 1, hazard ratio was adjusted for age and sex. In model 2, hazard ratio was adjusted for age, sex, low body weight, sequential organ failure assessment score, regular use of risk drugs for delirium before admission, performance status, Charlson comorbidity index, admission for central nervous system disorders, and pre-existing dementia.*

* *CI, confidence interval*.

### Association of Window Group With Secondary Outcomes

The unadjusted cumulative hazard curves for “delirium with event” and “delirium” within 30 days after admission are described in Supplementary Figure 1. Log-rank testing revealed no significant differences between the groups for “delirium with event” within 14 days after admission (*p* = 0.72) or “delirium” within 14 or 30 days after admission (*p* = 0.99 and 0.77, respectively). Multivariate analysis with a Cox regression model also identified no significant associations between the window group and “delirium with event” within 30 days after admission (adjusted HR 0.89, 95% CI 0.61–1.32, *p* = 0.56) or “delirium” within 14 or 30 days after admission (adjusted HR 0.97, 95% CI 0.71–1.34, *p* = 0.95 and adjusted HR 0.95, 95% CI 0.70–1.30, *p* = 0.76, respectively).

The adjusted odds ratios (ORs) of the window group for secondary outcomes were estimated using logistic regression models including hospital stay longer than 14 days (adjusted OR 1.19, 95% CI 0.95–1.49, *p* = 0.11), transfer to the ICU (adjusted OR 1.28, 95% CI 0.44–3.66, *p* = 0.64), and death during hospitalization (adjusted OR 1.18, 95% CI 0.85–1.63, *p* = 0.30). No significant relationships were observed between the window group and any outcome.

### Subgroup Analysis on the Association of Window Group With Primary Outcome

**Figure 4:**
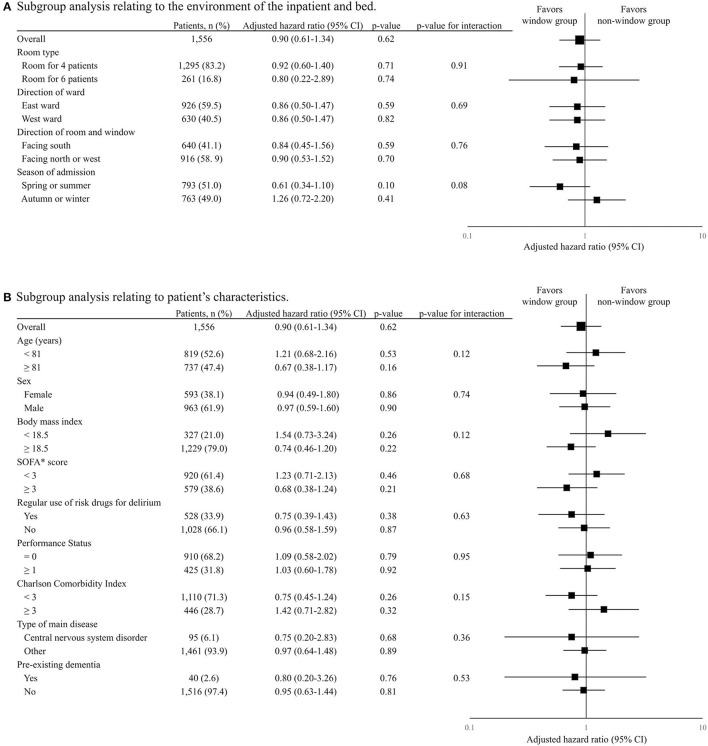
Subgroup analysis on the association of window group with primary outcome. The table and forest plot show the adjusted hazard ratio of the window group for delirium with event within 14 days after admission for each subgroup. The results of subgroup analyses in terms of inpatient environment and bed are shown in **(A)** and those regarding patient characteristics are shown in **(B)**. In the forest plot, black squares show the adjusted hazard ratio and horizontal lines show 95% CIs. Hazard ratio was modified using the Cox regression model adjusted for age, sex, low body weight, sequential organ failure assessment score, regular use of risk drugs for delirium before admission, performance status, Charlson comorbidity index, admission for central nervous system disorders, and pre-existing dementia.

The results of subgroup analysis are shown in Figure 4. No significant relationship was detected between the window group and the primary outcome in any subgroup regarding the inpatient environment or bed or patient characteristics. Similarly, no significant interaction effect was detected between the window group and any subgroup.

## Discussion

To the knowledge of the researchers, this is the first study to examine the effects of window-side bed assignment on delirium development in general wards. As several reports from the ICU found that rooms with a window could suppress delirium (21, 22, 40, 41), the admission to window-side beds in general wards was hypothesized to have a similar effect. However, no significant association was found between admission to a window-side bed and delirium development, even after adjusting for possible confounders.

Several reasons may explain why the expected association was absent in this investigation. First, the rooms were not compared with and without windows, but rather compared window-side beds with non-window-side beds. Although earlier studies reported the effect of the presence of a window to suppress delirium by comparing rooms with and without a window (21, 22, 40, 41), those results could have been caused not by the window itself, but by window-associated accompanying factors, such as the newness and clarity of the room. The present study compared window-side beds with non-window-side beds in identical layout rooms of a single center, which was considered to assess the direct impact of windows on delirium through sunlight exposure and visibility of the outside world. Indeed, almost half of the ICU research reported that windows did not decrease the development of delirium (42), implying the possibility that the simple presence of a window did not associate with delirium prevention. Furthermore, any significant associations for window-side beds were not compared with delirium in subgroup analyses factoring the environments of patients or rooms. These results support the result that windows do not directly impact delirium. Second, the illnesses of the patients in this study were considerably milder than those of previous reports. According to the ICU research, window placement suppressed delirium by maintaining cognitive function and efficient sleep (43). However, patients in general wards like those in this study have generally milder illnesses and less frequently experience cognitive dysfunction or sleeping disorders (44). Thus, it was possible that the cohort study did not require such window effects as cognitive function and sleep maintenance, which in turn did not appreciably reduce delirium development. Third, it was conceivable that the study's statistical power was insufficient to detect differences between the test groups. The risk difference in the ratio of the primary outcome was 0.6% (7.3% in the window group and 7.9% in the non-window group) in this study, which was considerably less than the predicted 5%. Thus, the effect of a window-side bed on delirium suppression was much smaller than estimation, and hence the sample size might not have been sufficient to detect an effect. However, considering the very small risk difference between the groups, window-side bed placement may not be the main deciding factor to prevent delirium development in the clinical setting. The bed placement of patients at higher risk of delirium may be better decided by such factors as patient request or proximity to a staff station rather than by window-side or not. Additional research with a larger sample size is needed for further assessment.

This study had several limitations that must be considered when interpreting the results. First, it was conducted retrospectively, and the patients were not allocated randomly to window or non-window groups. There was also the possibility that unmeasured confounding factors influenced the results. Indeed, some potential confounders could not be abstracted, including family structure of the patient, catheterization during hospitalization, whether the dividing curtain between beds was opened or closed, and patient bed location request. Moreover, the number of patients in the window group was much smaller than in the non-window group, even after considering the difference in numbers of window-side and non-window-side beds. Although it is assumed that this imbalance is due to the differences in hospitalization period and bed turnover rate between the groups, it cannot be denied that patient assignment was influenced by unmeasured confounders. Second, the diagnostic accuracy of delirium was presumed as not completely accurate because delirium development referring to medical charts was retrospectively abstracted. In retrospective studies on delirium, the chart abstraction method by Inouye et al. was generally used with a sensitivity and specificity of 73 and 84%, respectively (33). In the present research, however, diagnosis by this method may have had diminished accuracy since the reviewers were not delirium specialists. To enhance diagnostic accuracy, however, whether the abstracted “delirium” was accompanied by intervention was additionally assessed, and so “delirium with event” was abstracted as a more reproducible and specific outcome. Although this assessment method likely could not sufficiently abstract mild or hypoactive delirium, it was considered that “delirium with event” would be more suitable as the main outcome presuming that non-specialists of delirium basically observe, manage, and treat severe or hyperactive delirium cases more frequently than mild or hypoactive cases in general wards (45). The association of the window group was also evaluated with “delirium” defined by the chart abstraction method, and again no significant relationship was found, implying high reproducibility of this research. Lastly, the external validity of this research is limited due to its design as a single-center study at a university hospital. It is unclear whether the results can be applied to inpatients at other centers, especially those at long-term hospitals or nursing homes, because their characteristics and hospitalization environments differ considerably. Although the negative results in all subgroup analyses in this study partially support the external validity of the findings, additional multi-center studies will be necessary.

In summary, this study revealed no significant association between admission to a window-side bed and delirium development in older patients with a medical disease in a general ward. Clinically, the ideal bed placement of patients at higher risk of delirium may be more optimally decided by factors other than window-side location. Larger multi-center studies are warranted to refine and validate results.

## Data Availability Statement

The raw data supporting the conclusions of this article will be made available by the authors, without undue reservation.

## Ethics Statement

The studies involving human participants were reviewed and approved by Ethics Committee of Shinshu University Hospital. Written informed consent for participation was not required for this study in accordance with the national legislation and the institutional requirements.

## Author Contributions

DA has full access to all of the data in this research and takes responsibility for the integrity of the data and the accuracy of the data analysis. DA designed the study and drafted the article with support from YY and MH. DA and YY checked the medical charts and assessed the incidence of delirium. MH, KH, YY, and YK revised the article critically for important intellectual content and gave final approval of the submitted version. There are no contributors who should be listed other than the authors. All authors contributed to the article and approved the submitted version.

## Funding

This article was supported by a grant from the Shinshu Public Utility Foundation for Promotion of Medical Sciences.

## Conflict of Interest

The authors declare that the research was conducted in the absence of any commercial or financial relationships that could be construed as a potential conflict of interest.

## Publisher's Note

All claims expressed in this article are solely those of the authors and do not necessarily represent those of their affiliated organizations, or those of the publisher, the editors and the reviewers. Any product that may be evaluated in this article, or claim that may be made by its manufacturer, is not guaranteed or endorsed by the publisher.
