# Supplementary material for: Hospital Admission to a Window-Side Bed Does Not Prevent Delirium: A Retrospective Cohort Study of Older Medical Inpatients in General Wards
Source: Front Med (Lausanne). 2021 Sep 14;8:744581. doi: 10.3389/fmed.2021.744581 (PMC8476745; doi:10.3389/fmed.2021.744581)
Supplement: Supplementary file 1 [file Data_Sheet_1.docx]

Supplementary Material

# Supplementary Figure

**Supplementary Figure S1: Unadjusted cumulative curves for delirium with event and delirium**

**
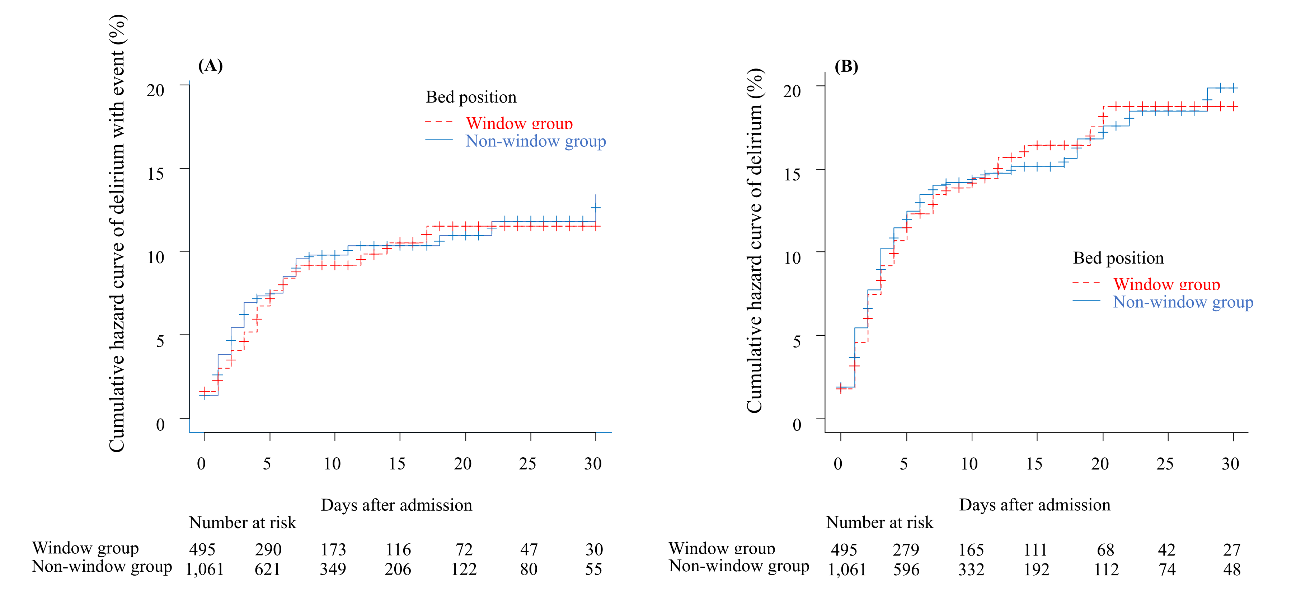
**

Figures show the cumulative Kaplan–Meier curves of delirium with event (A) and delirium (B) within 30 days after admission.
